# Association Between Macrophage Migration Inhibitory Factor -173 G>C Gene Polymorphism and Childhood Idiopathic Nephrotic Syndrome: A Meta-Analysis

**DOI:** 10.3389/fped.2021.724258

**Published:** 2021-10-15

**Authors:** Daojing Ying, Mengjie Jiang, Liping Rong, Hongjie Zhuang, Lizhi Chen, Yuanyuan Xu, Xiaoyun Jiang

**Affiliations:** Department of Pediatric Nephrology and Rheumatology, The First Affiliated Hospital, Sun Yat-sen University, Guangzhou, China

**Keywords:** MIF, gene polymorphism, idiopathic nephrotic syndrome, meta-analysis, children

## Abstract

**Background:** Studies have identified that MIF -173 G>C gene polymorphism is associated with idiopathic nephrotic syndrome (INS) susceptibility and steroid resistance, but the results remain inconclusive.

**Methods:** We searched PubMed, Embase, and Web of Science for relevant studies published before 31 March 2021. Pooled data were reported as odds ratio (OR) with 95% confidence interval (CI). Noteworthiness of significant OR was estimated by the false positive report probability (FPRP) test. Trial sequential analysis (TSA) was used to control type I and type II errors.

**Results:** We selected seven case-control studies that included 1,026 INS children (362 were steroid-resistant NS and 564 were steroid-sensitive NS) and 870 controls. The results showed that MIF -173 G>C polymorphism was significantly associated with INS susceptibility in allelic, heterozygous and dominant genetic models (C vs. G: OR = 1.325, 95% CI: 1.011-1.738; GC vs. GG: OR = 1.540, 95% CI: 1.249-1.899; CC + GC vs. GG: OR = 1.507, 95% CI: 1.231-1.845), and FPRP test and TSA indicated that the associations were true in heterozygous and dominant models. The pooled results also revealed that MIF -173 G>C polymorphism was significantly associated with steroid resistance in allelic, homozygous and recessive models (C vs. G: OR = 1.707, 95% CI: 1.013-2.876; CC vs. GG: OR = 4.789, 95% CI: 2.109-10.877; CC vs. GC + GG: OR = 4.188, 95% CI: 1.831-9.578), but FPRP test indicated that all these associations were not noteworthy. Furthermore, TSA revealed that the non-significant associations between MIF -173 G>C polymorphism and steroid resistance in heterozygous and dominant models were potential false negative.

**Conclusions:** This meta-analysis could draw a firm conclusion that MIF -173 G>C polymorphism was significantly associated with increased INS risk in heterozygous and dominant genetic models. MIF -173 G>C polymorphism was not likely to affect steroid responsiveness, but more studies were needed to confirm.

## Introduction

Childhood idiopathic nephrotic syndrome (INS) is characterized by severe proteinuria, hypoalbuminemia, and generalized edema. It is one of the most common glomerular diseases with an incidence of 1·15-16·9 per 100,000 children (1). Glucocorticoids (GCs) are the first-line treatment regimens for INS and induce complete remission (CR) in most patients, who are therefore diagnosed with steroid-sensitive nephrotic syndrome (SSNS). In contrast, approximately 20% of patients who do not achieve CR after the initial standard course of GCs are diagnosed with steroid-resistant nephrotic syndrome (SRNS) and at high risk for progression to ESRD (1). The exact pathogenesis of INS and the precise mechanism of the difference in steroid response remain unknown. Current evidence indicates that genetic polymorphisms can affect INS susceptibility and predict steroid treatment response (2, 3).

Macrophage migration inhibitory factor (MIF) is a pleiotropic cytokine that contributes to the pathogenesis of many immune-inflammatory and autoimmune diseases and can also regulate glucocorticoid-mediated immunosuppression (4, 5). Two functional promotor gene polymorphisms in MIF, namely the -794 CATT_5−8_ microsatellite repeat (rs5844572) and the -173 G>C (rs755622), are associated with disease susceptibility or clinical severity (4, 5). Recently, growing studies focus on the association between MIF -794 CATT_5−8_ or -173 G>C polymorphism and INS susceptibility as well as steroid treatment response. At present, a total of two studies investigated the association between MIF -794 CATT_5−8_ polymorphism and INS, and both reported that a higher CATT number was not associated with increased risk of INS and steroid resistance (3, 6). However, studies related to the association between MIF -173 G>C polymorphism and INS presented conflicting results, so we conducted a meta-analysis to clarify the association between the MIF -173 G>C polymorphism and INS.

## Materials and Methods

### Exposure and Outcomes

In this meta-analysis, exposure is G to C transition at position -173 of MIF gene, and outcomes are INS and steroid resistance.

### Search Strategy

Two independent researchers (DJY and HJZ) comprehensively searched PubMed, Embase and Web of Science to identify eligible studies regarding the association between MIF -173 G>C polymorphism and INS published up to 31 March 2021. The search strategy was (“macrophage migration inhibitory factor” or MIF) and (polymorphism or mutation or variant or genotype) and “nephrotic syndrome.” In addition, references from relevant articles and reviews were also searched for additional worthy studies. The search was restricted to English language papers. Discrepancies were resolved by a third researcher (MJJ) or consensus-based discussion.

### Inclusion and Exclusion Criteria

The following inclusion criteria were used to select the eligible studies: (1) case-control studies investigated the association between MIF -173 G>C polymorphism and childhood INS (onset age <18 years); (2) studies have enough data to calculate odds ratios (ORs) and 95% confidence intervals (CIs). The exclusion criteria were: (1) study included secondary cause of NS; (2) case reports, meeting abstract, reviews, repeated literature; (3) detailed genotype data were not available. Furthermore, for the meta-analysis of MIF -173 G>C polymorphism and steroid resistance, studies that did not classify INS patients into SSNS and SRNS were excluded.

### Data Extraction and Quality Assessment

Data extracted from individual studies include: the first author's name, year of publication, country, sample size, genotyping methods, diagnostic criteria of NS, SRNS and SSNS, frequencies of genotypes and the value of Hardy-Weinberg equilibrium (HWE) test. For each study, HWE was calculated using a web-based statistical tool (https://ihg.gsf.de/cgi-bin/hw/hwa1.pl). We used the Newcastle-Ottawa scale (NOS) (7) to assess the methodological quality of eligible studies and defined a score of 6 or higher as high study quality. Two reviewers (LPR and LZC) conducted the data analysis with any disagreements resolved by discussion and consensus.

### Statistical Analysis

We used Stata 14.0 (Stata Corp, College Station, TX) to perform data analysis. Pooled ORs and 95% CIs were used to assess the association of MIF -173 G>C polymorphism with INS susceptibility and steroid responsiveness. The pooled ORs were computed for the allelic (C vs. G), homozygous (CC vs. GG), heterozygous (GC vs. GG), dominant (CC + GC vs. GG) and recessive (CC vs. GC + GG) genetic models. The statistical significance of the pooled ORs was determined with the *Z*-test, and *P* < 0.05 was considered significant. Heterogeneity among the studies was checked using the *Q*-test and *I*^2^ test, with *I*^2^ > 50% indicating high heterogeneity. The fixed-effect model was selected to calculate pooled ORs when *I*^2^ < 50% or *P*_Q_ > 0.1 (8). Otherwise, the random-effect model was used. Sensitivity analysis was conducted to check the stability of overall pooled ORs by excluding one study at a time. Publication bias was evaluated by drawing the Funnel plot and further confirmed by Egger's and Begg's tests (*P* < 0.05 was considered significant publication bias).

We performed false-positive report probability (FPRP) tests for all the significant pooled ORs. FPRP values were calculated at the prior probability of 0.001 to detect ORs of 1.5 by using the Excel spreadsheet offered by Wacholder et al. (9). An FPRP < 0.2 was considered as a noteworthy association. In addition, we conducted the trial sequential analysis (TSA) to control type I and type II errors, estimated the required information size (RIS) base on 5% probability of a type I error (two-sided α = 0.05), a statistical test power of 80% (type II error rate = 0.2) and a relative risk reduction (RRR) of 20% (10, 11). If the cumulative Z-curve crosses the trial sequential monitoring boundary with or without exceeding the RIS line, it means that the statistically significant result is a true positive, with no further studies required (11). If the cumulative Z-curve does not cross the futility boundary and does not reach the RIS line, the non-significant result is a potential false negative, and more studies are needed (11, 12). TSA was performed using Trial Sequential Analysis software version 0.9.5.10 β (available at www.ctu.dk/tsa).

## Results

### Study Characteristics

**Figure 1:**
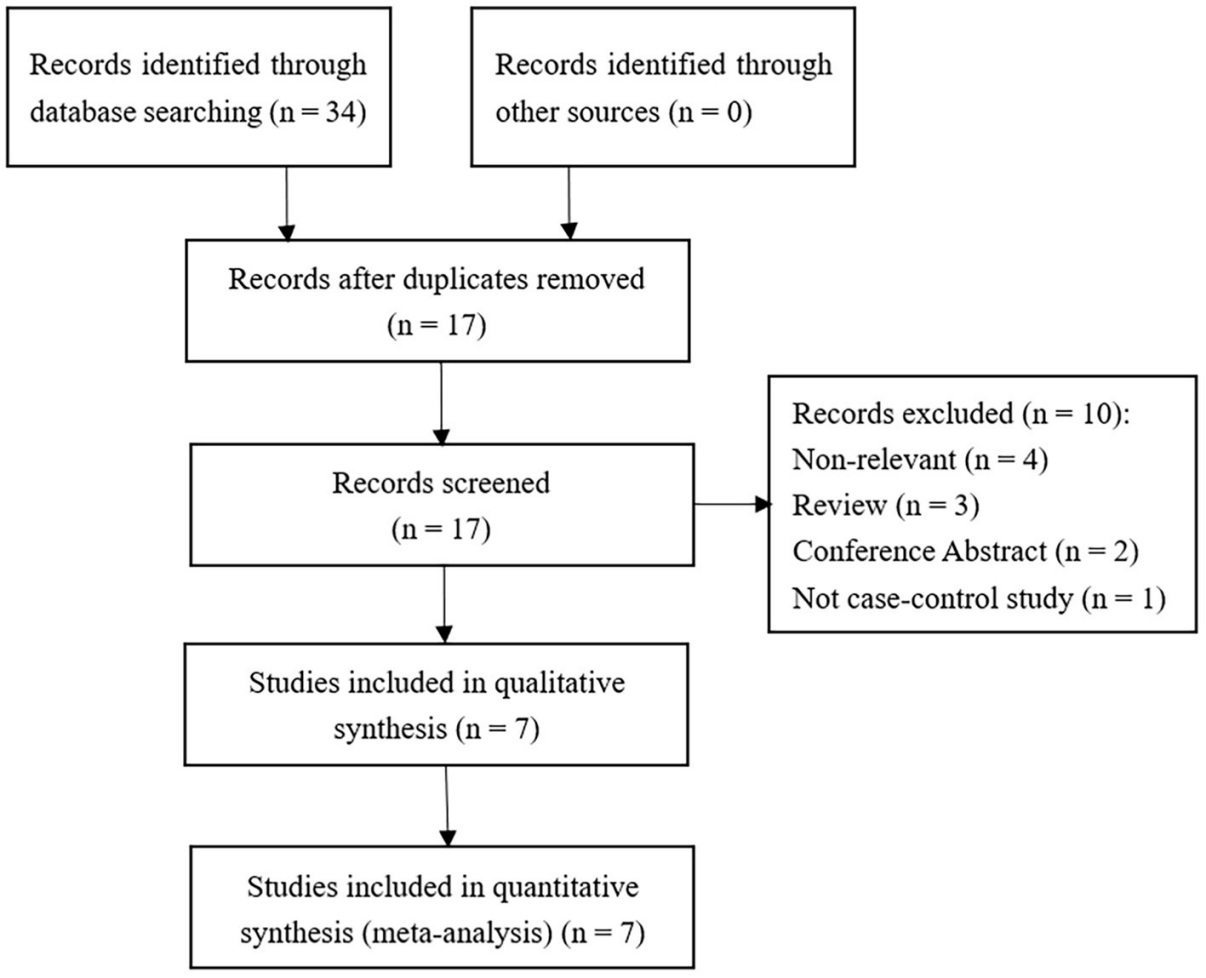
Flow diagram for study inclusion.

According to the literature search strategy and selection criteria, seven studies were identified as shown in Figure 1. 1,026 INS patients (564 cases diagnosed with SSNS and 362 cases diagnosed with SRNS) and 870 control patients were enrolled in this meta-analysis. One study did not explicitly classify INS into SSNS and SRNS according to steroid responsiveness (13), so we excluded it in the meta-analysis of MIF -173 G>C polymorphism and steroid responsiveness. In the other six studies, three studies provided a specific definition of steroid resistance (14–16). According to the NOS scale, all the included studies were considered to be of high quality (score ≥ 6). In all the included studies, the control group of one study did not conform to HWE (*P* < 0.05) (14). The main characteristics of the included studies are present in Table 1. The information of age, gender, and definitions of INS and steroid resistance is present in Supplementary Table S1.

**Table 1:** Main characteristics of eligible studies.

**Study**	**Country**	**Genotyping methods**	**Case (SSNS, SRNS)**			**Control**			*P*_HWE_ ^*^	**NOS score**
			**CC**	**CG**	**GG**	**CC**	**CG**	**GG**		
Berdeli et al. (14)	Turkey	PCR-RFLP	11 (1, 10)	60 (30, 30)	143 (106, 37)	3	12	88	0.03	7
Vivarelli et al. (15)	Italy	PCR- DHPLC	2 (0, 2)	79 (34, 45)	176 (115, 61)	2	75	278	0.20	7
Choi et al. (17)	Korea	PCR-RFLP	2 (1, 1)	59 (33, 26)	109 (67, 42)	3	32	65	0.70	6
Swierczewska et al. (6)	Poland	PCR Sequencing	4 (2, 2)	24 (13, 11)	43 (15, 28)	1	13	16	0.39	6
Ramayani et al. (16)	Indonesia	PCR-RFLP	5 (1, 4)	33 (11, 22)	42 (28, 14)	3	13	24	0.52	7
Suvanto et al. (13)	Finland	PCR Sequencing	5 (–, –)	34 (–, –)	61 (–, –)	9	29	63	0.05	6
Sadeghi-Bojd et al. (18)	Iran	PCR-RFLP	5 (3, 2)	49 (40, 9)	80 (64, 16)	2	35	104	0.62	8

*SSNS, steroid-sensitive nephrotic syndrome; SRNS, steroid-resistant nephrotic syndrome; PCR, Polymerase chain reaction; RFLP, Restriction fragment length polymorphism; DHPLC, Denaturing high-performance liquid chromatography; NOS, Newcastle Ottawa Scale; HWE, Hardy-Weinberg equilibrium;*

* *P_HWE_ for the control groups*.

### Association Between MIF -173 G > C Polymorphism and INS Susceptibility

**Figure 2:**
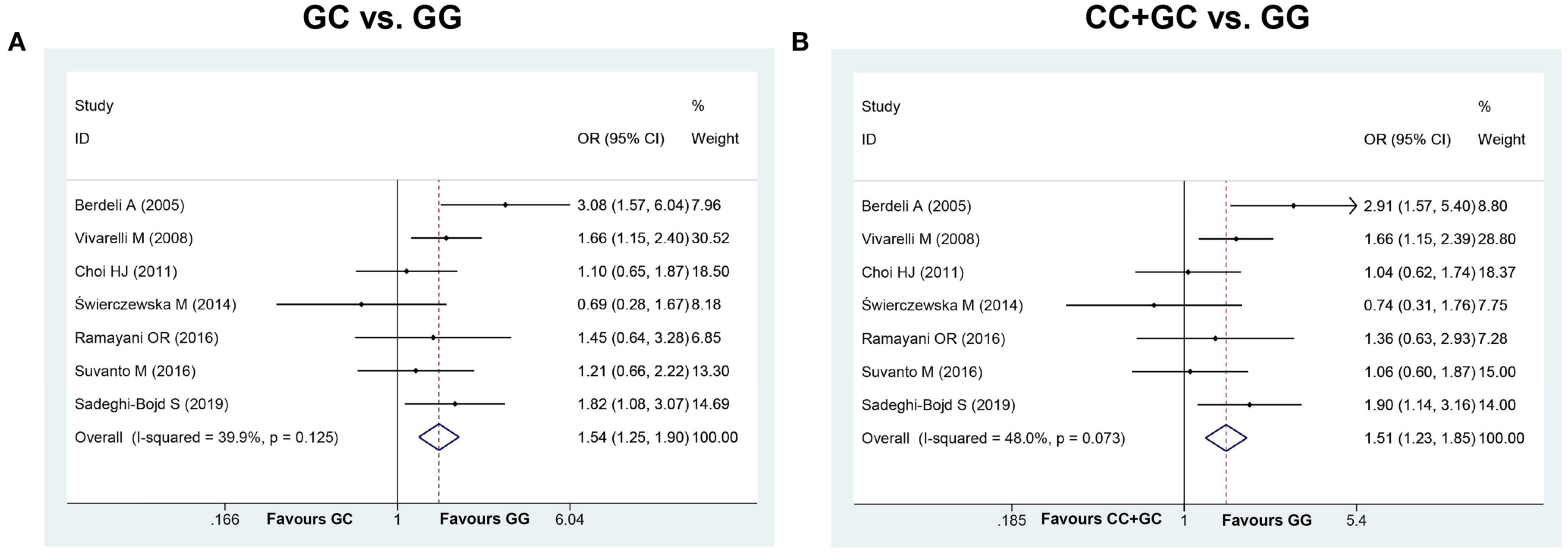
Forest plots (**A**: heterozygous model; **B**: dominant model) for the association between MIF -173 G > C polymorphism and INS susceptibility.

**Figure 3:**
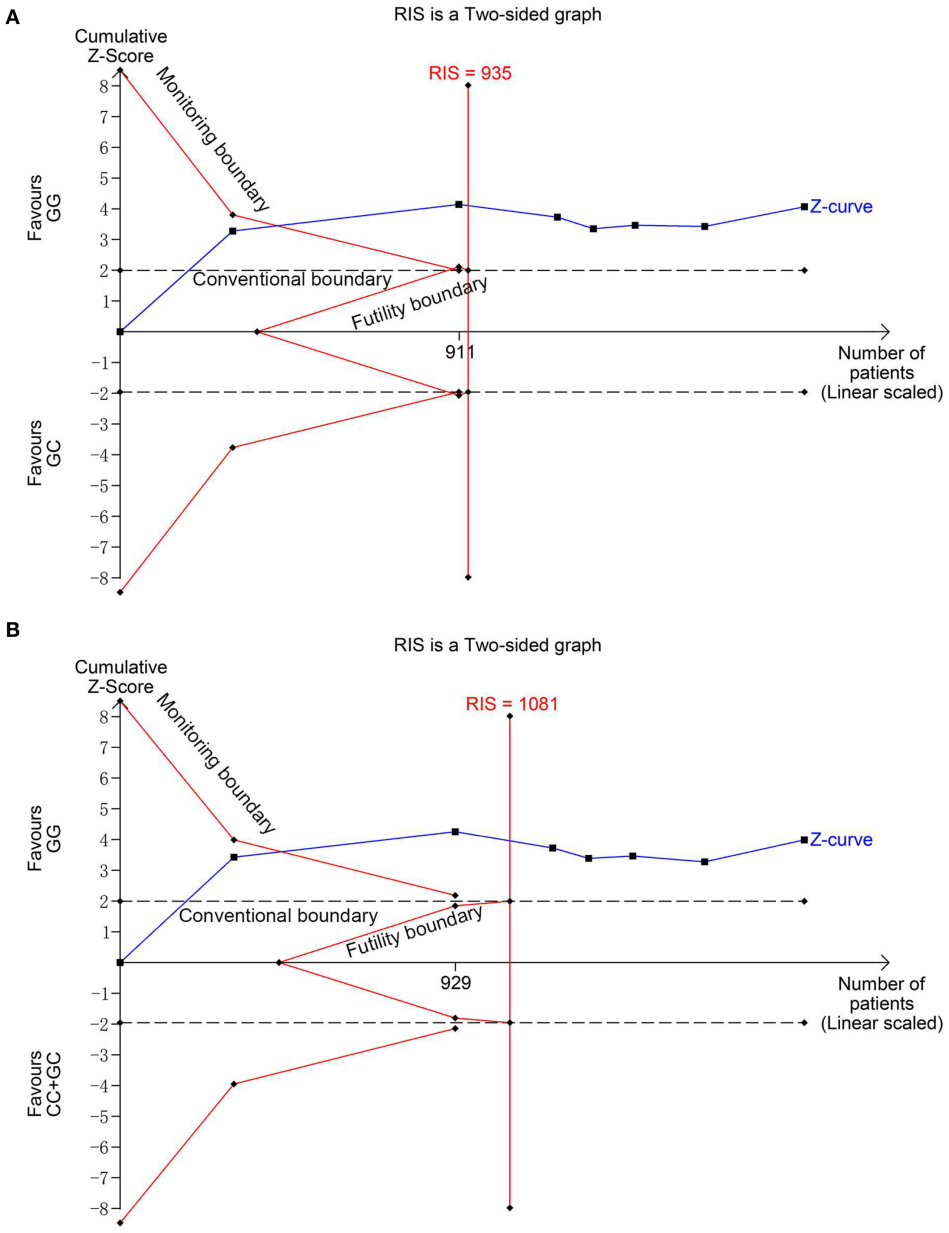
Trial sequential analysis for the association between MIF -173G > C polymorphism and INS susceptibility under the heterozygous **(A)** and dominant **(B)** models. The required information size (RIS) was calculated based on α = 5%, 80% power, and a relative risk reduction of 20%. The cumulative Z-curves crossed the monitoring boundaries and surpassed the RIS in **(A)** and **(B)**, indicating sufficient evidence for the significant associations.

Seven studies (6, 13–18) involving 1,026 cases and 870 controls were included in this meta-analysis. The pooled results showed MIF -173 G>C polymorphism was significantly associated with increased INS risk in three genetic models, i.e., allelic (C vs. G: OR = 1.325, 95% CI: 1.011-1.738, *P* = 0.042), heterozygous (GC vs. GG: OR = 1.540, 95% CI: 1.249-1.899, *P* < 0.001), and dominant model (CC + GC vs. GG: OR = 1.507, 95% CI: 1.231-1.845, *P* < 0.001) (Table 2, Supplementary Figures S1A, Figures 2A,B). These statistically significant associations were investigated by using the FPRP test. At the pre-specified prior probability level of 0.001 to detect OR of 1.50, the FPRP values for the association of MIF -173 G>C polymorphism and INS susceptibility in allelic, heterozygous and dominant models were 0.981, 0.118, and 0.129, respectively (Table 2). Therefore, the associations were found noteworthy (FPRP <0.2) only in heterozygous and dominant models. TSA was further performed to evaluate the reliability of the meta-analysis results in the two genetic models. For MIF -173 G>C polymorphism and INS susceptibility in heterozygous and dominant models, the cumulative Z-curve crossed the monitoring boundary and surpassed the required information size, indicating the associations were statistically significant adjusted for multiple comparisons and the sample sizes were adequate (Figures 3A,B). Furthermore, there was no obvious heterogeneity among the studies in heterozygous and dominant models (Table 2, Figures 2A,B). Sensitivity analysis showed that the meta-analysis results were relatively stable in heterozygous and dominant models (Supplementary Figures S2A,B). There was also no statistically significant publication bias in heterozygous and dominant models (Table 2, Supplementary Figures S2C,D).

**Table 2:** Summary of the association between MIF -173 G > C polymorphism and INS susceptibility.

**Comparisons**	**Test of association**		**Test of heterogeneity**		**Effects model**	**FPRP value of different prior probability**			**Begg's test**	**Egger's test**
	**OR (95%CI)**	***P***	**I^**2**^%**	***P***		**0.1**	**0.01**	**0.001**		
C vs. G	1.325 (1.011, 1.738)	0.042	53.9	0.043	Random	0.317	0.836	0.981	0.764	0.601
CC vs. GG	1.172 (0.671, 2.046)	0.577	0.0	0.486	Fix				0.764	0.649
GC vs. GG	1.540 (1.249, 1.899)	<0.001	39.9	0.125	Fix	**0.001**	**0.013**	**0.118**	0.548	0.562
CC + GC vs. GG	1.507 (1.231, 1.845)	<0.001	48.0	0.073	Fix	**0.001**	**0.014**	**0.129**	0.548	0.497
CC vs. GC + GG	1.040 (0.598, 1.809)	0.889	0.0	0.569	Fix				0.548	0.505

*OR, odds ratio; CI, confidence interval; FPRP, false positive report probability*.

*FPRP values < 0.2 were bold*.

### Association Between MIF -173 G > C Polymorphism and Steroid Responsiveness

**Figure 4:**
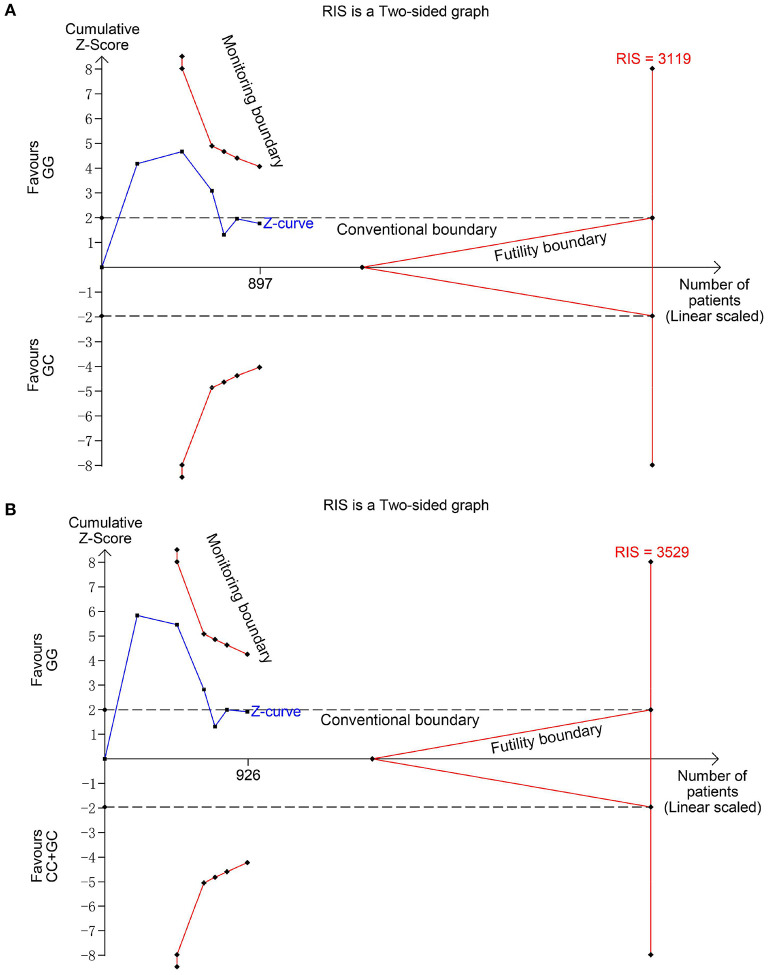
Trial sequential analysis for the association between MIF -173 G > C polymorphism and steroid responsiveness under the heterozygous **(A)** and dominant **(B)** models. The required information size (RIS) was calculated based on α = 5%, 80% power, and a relative risk reduction of 20%. The cumulative Z-curves did not cross monitoring and futility boundaries and also did not reach the RIS in **(A)** and **(B)**, indicating the results were inconclusive and more studies were needed.

Six studies (6, 14–18) including 564 SSNS patients and 362 SRNS patients focused on the association between MIF -173 G > C polymorphism and steroid responsiveness, and the meta-analysis showed significantly increased risk of steroid resistance in allelic, homozygous, and recessive models (C vs. G: OR = 1.707, 95% CI: 1.013-2.876, *P* = 0.044; CC vs. GG: OR = 4.789, 95% CI: 2.109-10.877, *P* < 0.001; CC vs. GC + GG: OR = 4.188, 95% CI: 1.831-9.578, *P* = 0.001) (Table 3, Supplementary Figures S3A-C). However, all these significant associations were not noteworthy under the FPRP test (all FPRP > 0.2, Table 3). TSA was performed to control type II error for the non-significant associations between MIF -173 G > C polymorphism and steroid responsiveness in heterozygous and dominant models (Figures 4A,B). The cumulative Z-curves did not cross monitoring boundaries and futility boundaries and also did not reach the required information size. Thus, the results were potential false negative in heterozygous and dominant models, and more studies were needed to conduct. Sensitivity analysis showed stable results in allelic, heterozygous and dominant models (Supplementary Figures S4A,D,E). However, in homozygous and recessive models, when the study by Berdeli et al. was excluded, no significant association with steroid resistance risk was found (Supplementary Figures S4B,C). Egger's test and Begg's test reflected that no statistical significance of publication bias was detected in the five genetic models (all *P* > 0.05) (Table 3).

**Table 3:** Summary of the association between MIF -173 G > C polymorphism and INS steroid responsiveness.

**Comparisons**	**Test of association**		**Test of heterogeneity**		**Effects Model**	**FPRP value of different prior probability**			**Begg's test**	**Egger's test**
	**OR (95%CI)**	***P***	**I^**2**^%**	***P***		**0.1**	**0.01**	**0.001**		
C vs. G	1.707 (1.013, 2.876)	0.044	77.3	0.001	Random	0.561	0.934	0.993	0.452	0.249
CC vs. GG	4.789 (2.109, 10.877)	<0.001	41.3	0.130	Fix	0.372	0.867	0.985	1.000	0.968
GC vs. GG	1.643 (0.942, 2.868)	0.080	69.9	0.005	Random				0.452	0.332
CC + GC vs. GG	1.780 (0.977, 3.243)	0.060	75.7	0.001	Random				0.260	0.341
CC vs. GC + GG	4.188 (1.831, 9.578)	0.001	16.6	0.307	Fix	0.453	0.901	0.989	1.000	0.892

*OR, odds ratio; CI, confidence interval; FPRP, false positive report probability*.

## Discussion

In this first meta-analysis aimed to investigate the associations between MIF -173 G>C polymorphism and INS susceptibility and steroid responsiveness, we performed the FPRP test and TSA to evaluate the results from the traditional pooled analysis method. Our results showed that MIF -173 G>C polymorphism was significantly associated with increased INS risk under both heterozygous and dominant models and the currently available evidence was sufficient to draw this conclusion. For MIF -173 G>C polymorphism and steroid responsiveness, no truly significant association was identified, and the currently available evidence was not enough to confirm the negative results.

MIF is a pleiotropic protein that acts as a potent upstream regulator of the immune system (5). Due to its proinflammatory role, MIF was proposed as a potential therapeutic target for autoimmune and inflammatory diseases (4). Studies have revealed that the promoter polymorphism at MIF -173 (G>C, rs755662) is associated with high levels of circulating MIF and increased disease susceptibility and severity in patients with systemic lupus erythematosus, rheumatoid arthritis, etc (5, 19–21). Thus researchers suggest that anti-MIF therapies may be most effective in patients with high-expression MIF genotype (5). Immune dysregulation plays a crucial role in the pathogenesis of most INS patients (22). Cuzzoni et al. observed that plasma MIF levels are significantly higher in INS patients before steroid treatment than in healthy controls (23). The present meta-analysis results showed the significant associations between the MIF -173 G>C polymorphism and INS susceptibility in heterozygous and dominant models, which were confirmed by the FPRP test and TSA. In addition, there was no obvious heterogeneity and publication bias under the two models. Sensitivity analysis also indicated that the pooled results were stable in heterozygous and dominant models. Therefore, we could draw a firm conclusion that MIF -173 G>C polymorphism was significantly associated with increased risk of childhood INS, and the heterozygous and dominant models were likely to be the most appropriate models to estimate risk. At present, steroids are still the mainstay of therapy for childhood INS and can cause a series of severe adverse effects such as osteoporosis, impaired growth and ocular complications (24). Anti-MIF therapies may be a potential choice in INS patients with high-expression MIF genotypes, and further research focuses on the relationship between MIF and pathogenesis of INS is meaningful.

MIF is also involved in the mechanism of resistance to steroids in autoimmune and inflammatory diseases (4, 5). MIF can antagonize glucocorticoid-mediated immunosuppression *via* affecting NF-κB/IκB signaling or triggering the mitogen-activated protein kinase (MAPK) signaling (5, 25, 26). Accordingly, MIF promoter polymorphisms were associated with clinically defined steroid resistance in autoimmune and inflammatory diseases such as juvenile idiopathic arthritis, inflammatory bowel disease and sudden sensorineural hearing loss (27–29). However, the association between MIF -173 G>C polymorphism and steroid resistance in INS is the most studied, but the results are not consistent. This meta-analysis showed significant associations between MIF -173 G>C polymorphism and steroid resistance in allelic, homozygous, and recessive models but not confirmed by the FPRP test. And sensitivity analysis also showed the pooled results were not stable in homozygous and recessive models. Besides, TSA showed that the non-significant associations in heterozygous and dominant models were potential false negative, and more studies are needed to reach the required information size. Tong et al. performed a meta-analysis focusing on the association between MIF -173 G>C polymorphism and renal disease susceptibility in 2015; they also pooled the data of three studies (6, 14, 15) to investigate the association between MIF -173 G>C polymorphism and steroid responsiveness of INS and concluded that the polymorphism was associated with increased risk of glucocorticoid resistance in childhood INS (30). The present meta-analysis updated the information and drew a different conclusion that MIF -173 G>C polymorphism was not likely to be truly associated with steroid resistance and more studies were needed. SRNS is currently considered a heterogeneous disease with complicated pathogenesis, various pathological types, and different response to immunosuppressants (31). Furthermore, the incidence of SRNS is significantly lower than that of SSNS (1). Thus, studies focused on the association between MIF promoter polymorphisms and steroid responsiveness in childhood INS should be well-designed.

There were some limitations in the meta-analysis. First, although the Begg's and Egger's tests did not reveal any statistical evidence of publication bias, selection bias might exist because only published studies were included. Second, we did not perform subgroup analysis based on ethnicity, because the number of included studies is small and lack of ethnic diversity as well as the ethnicity of several studies could not be clearly defined. Finally, researchers used different definitions or did not clearly report which definition was used could increase the risk of bias and contribute to heterogeneity, and the different percentages of SRNS among studies also infer selection bias might exist, and well-designed studies to investigate the association between MIF promoter polymorphisms and steroid responsiveness of childhood INS are needed.

In conclusion, the current meta-analysis could draw a firm conclusion that MIF -173 G>C polymorphism significantly increased the risk of childhood INS under the heterozygous and dominant genetic models in the overall population. Anti-MIF therapies might have potential value in childhood INS. The MIF -173 G>C polymorphism was not likely to truly increase the risk of steroid resistance in childhood INS, but further well-designed studies focused on this polymorphism and steroid responsiveness of INS are warranted to draw a firm conclusion.

## Data Availability Statement

The original contributions presented in the study are included in the article/Supplementary Material, further inquiries can be directed to the corresponding author/s.

## Author Contributions

XJ and YX designed the study. DY, MJ, LR, HZ, and LC performed the data extraction and analyses. DY, MJ, LR, and HZ drafted and revised the paper. All authors approved the final version.

## Funding

This work was supported by the Guangdong Basic and Applied Basic Research Foundation (Grant no. 2019A1515010694 to XJ and 2019A1515011546 to MJ), the Sun Yat-sen University Basic Scientific Research Young Teacher Training Project (Grant no. 19ykpy65 to YX), and the Science and Technology Planning Project of Guangzhou, China (Grant No. 202103000001 to XJ).

## Conflict of Interest

The authors declare that the research was conducted in the absence of any commercial or financial relationships that could be construed as a potential conflict of interest.

## Publisher's Note

All claims expressed in this article are solely those of the authors and do not necessarily represent those of their affiliated organizations, or those of the publisher, the editors and the reviewers. Any product that may be evaluated in this article, or claim that may be made by its manufacturer, is not guaranteed or endorsed by the publisher.
