# Supplementary material for: Association Between Macrophage Migration Inhibitory Factor -173 G>C Gene Polymorphism and Childhood Idiopathic Nephrotic Syndrome: A Meta-Analysis
Source: Front Pediatr. 2021 Oct 15;9:724258. doi: 10.3389/fped.2021.724258 (PMC8555679; doi:10.3389/fped.2021.724258)

Supplementary Material

1. **Supplementary Tables**

**Table S1** Age, gender, and definitions of INS and steroid resistance in the included studies

| Study | Age at INS onset (mean ± SD, years) | Male (%) | Definition of INS | Definition of steroid resistance |
| --- | --- | --- | --- | --- |
| Berdeli A (14) | 3.5 ± 2.9 | 59.3 | ISKDC criteria | No achievement of remission with prednisolone at 2 mg/kg per day for 4 weeks |
| Vivarelli M (15) | 5.8 ± 4.2 | 59.1 | Not specified | No achievement of remission with prednisolone at 60 mg/m^2^/day for 6-8 weeks |
| Choi HJ (16) | 5.17 ± 3.31 | 70.0 | Massive proteinuria of ≥ 40 mg/h/m^2^ with hypoalbuminemia of ≤ 2.5 g/dL without known causes | Not specified |
| Świerczewska M (6) | 10.1 ± 4.4 | 59.2 | Not specified | Not specified |
| Ramayani OR (17) | SR: 5.9 ± 3.3, SS: 6.2 ± 3.2 | 68.8 | Not specified | A failure to achieve complete remission following 6–8 weeks of daily 60 mg/m^2^ steroid treatment or following 4 weeks of daily 60 mg/m^2^ steroid treatment and administration of three pulses of methylprednisolone |
| Suvanto M (13) | ＜3y: 19.8%; ＞3y: 80.2% | Not Available | ISKDC criteria | Not specified |
| Sadeghi-Bojd S (18) | 5.5 ± 2.8 | 60.0 | Not specified | Not specified |

INS, idiopathic nephrotic syndrome; SD, standard deviation; ISKDC, International Study of Kidney Disease in

Children

1. **Supplementary Figures**

**Figure S1** Forest plots (A: allelic model; B: homozygous model; C: recessive model) for the association between MIF -173 G>C polymorphism and INS susceptibility.


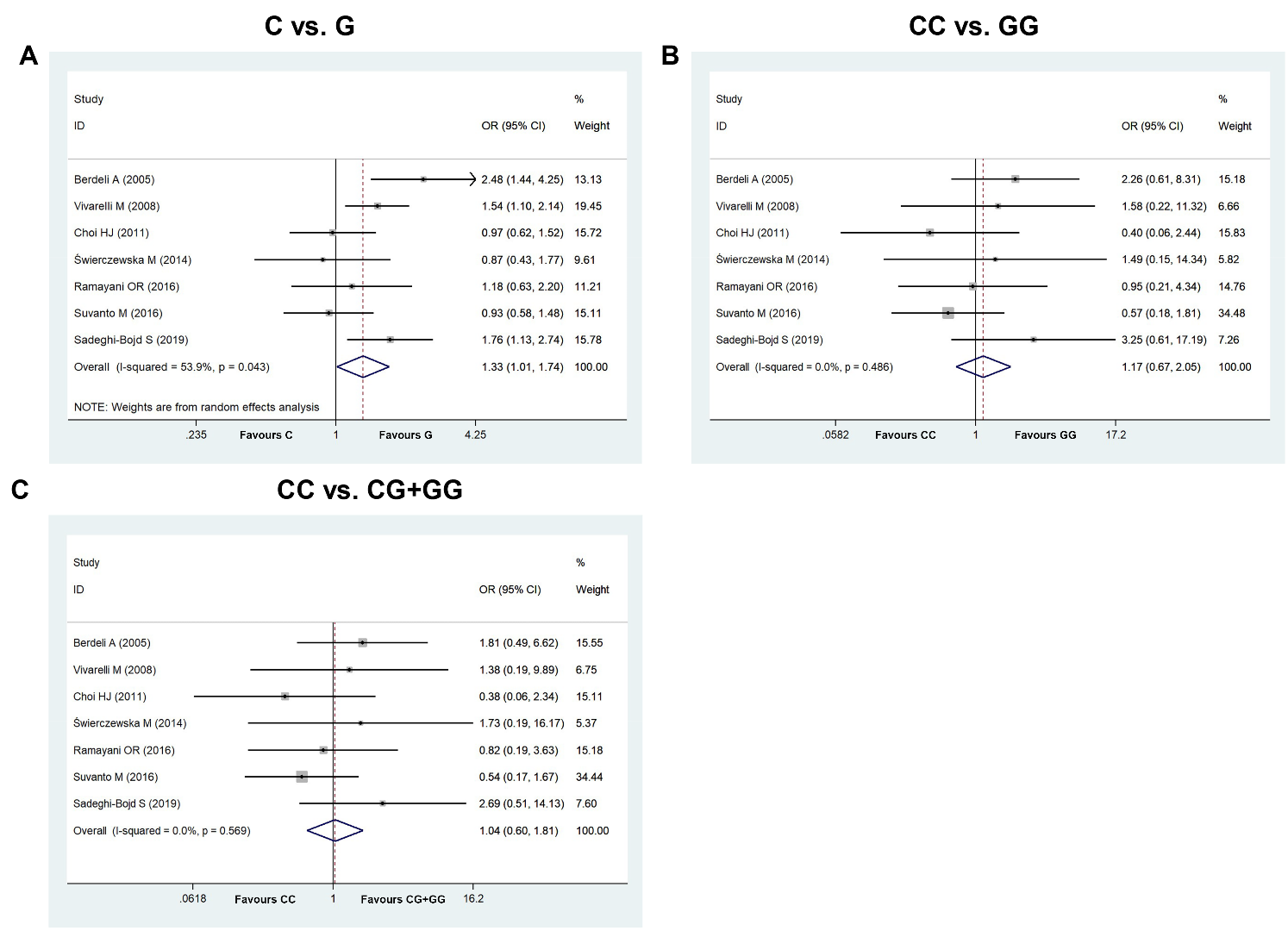


**Figure S2** Sensitivity analyses (A: heterozygous model; B: dominant model) and funnel plots (C: heterozygous model; D: dominant model) for the association between MIF -173 G>C polymorphism and INS susceptibility.


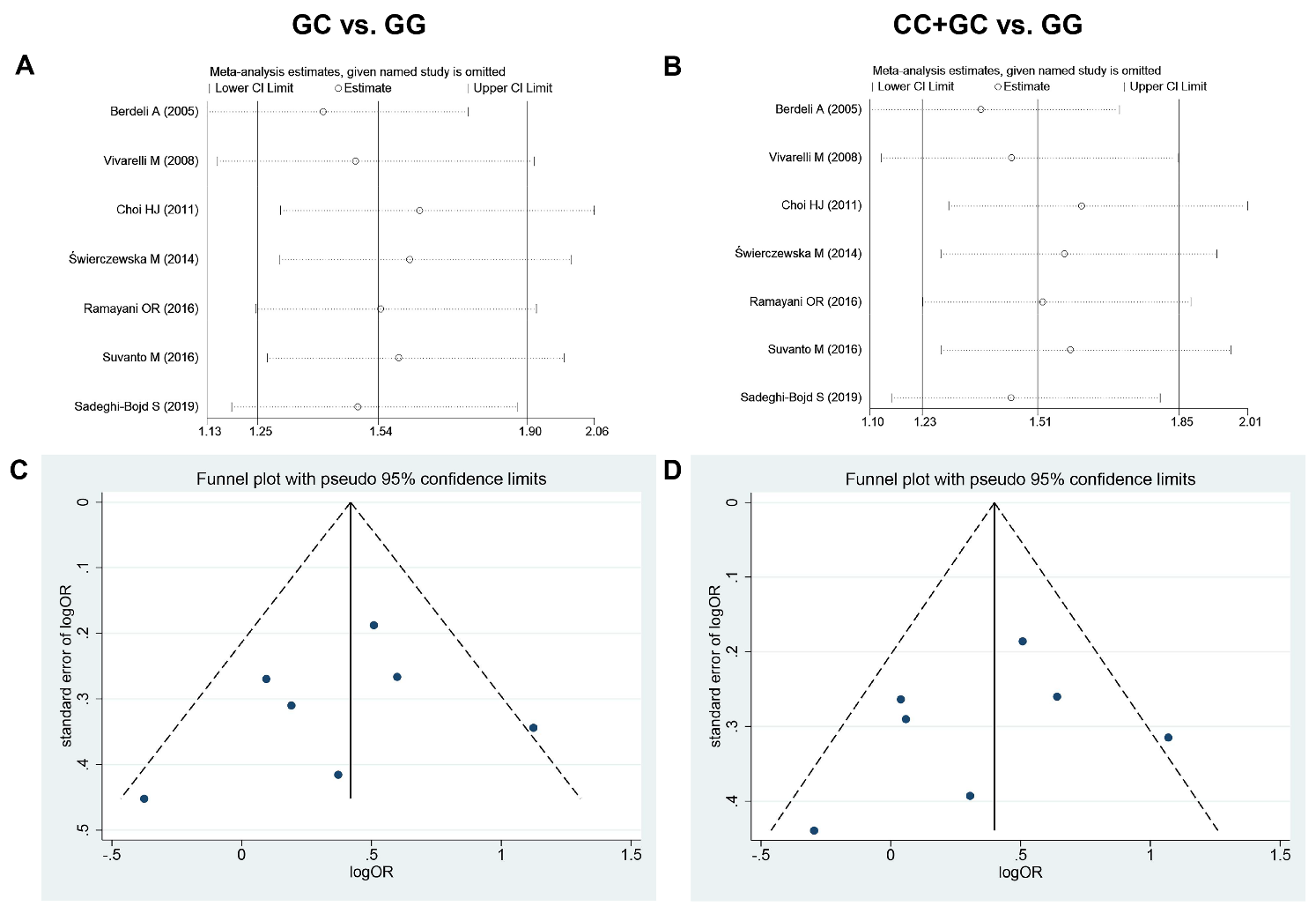


**Figure S3** Forest plots (A: allelic model; B: homozygous model; C: recessive model; D: heterozygous model; E: dominant model) for the association between MIF -173 G>C polymorphism and steroid responsiveness.


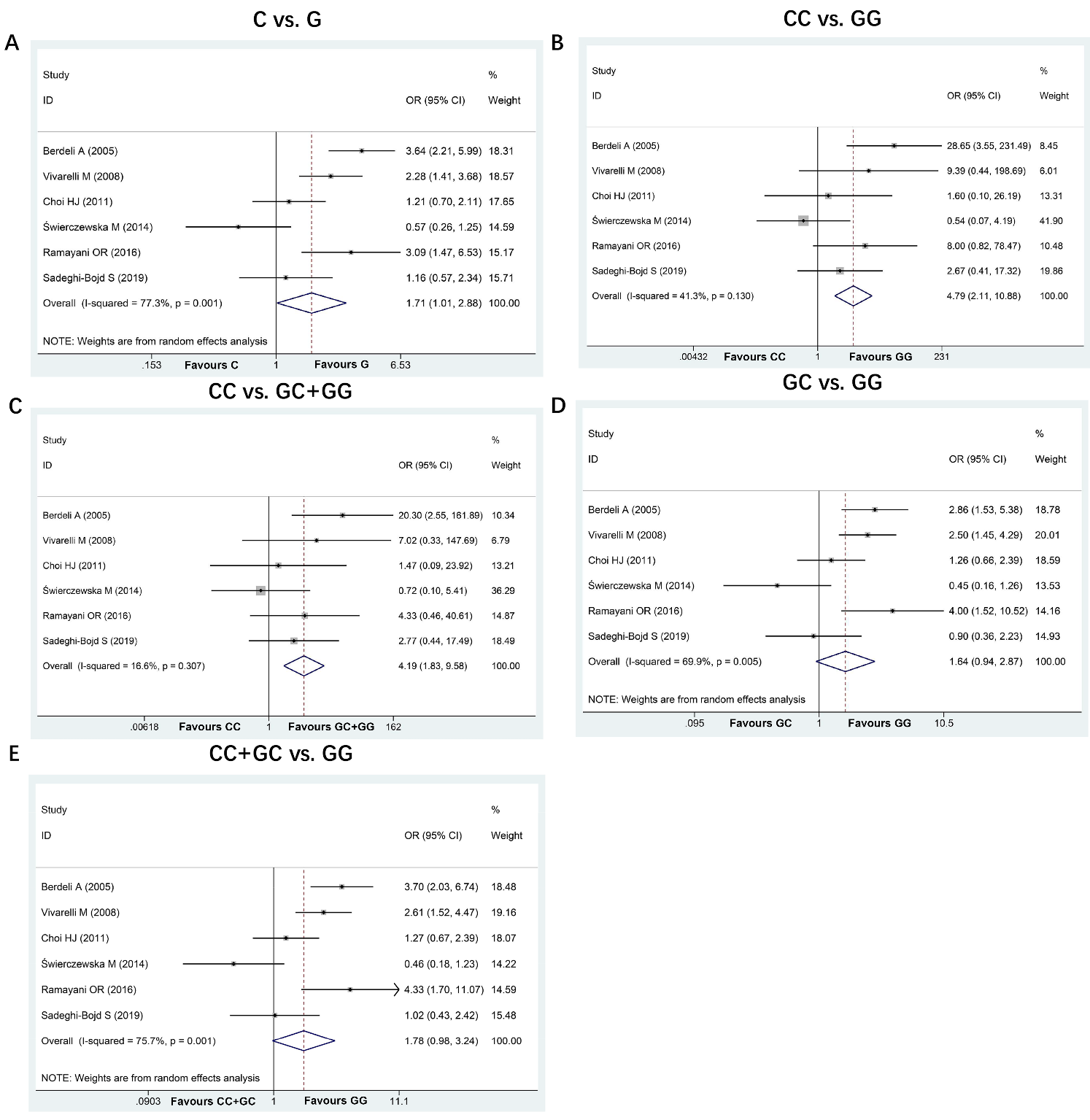


**Figure S4** Sensitivity analyses (A: allelic model; B: homozygous model; C: recessive model; D: heterozygous model; E: dominant model) for the association between MIF -173 G>C polymorphism and steroid responsiveness.


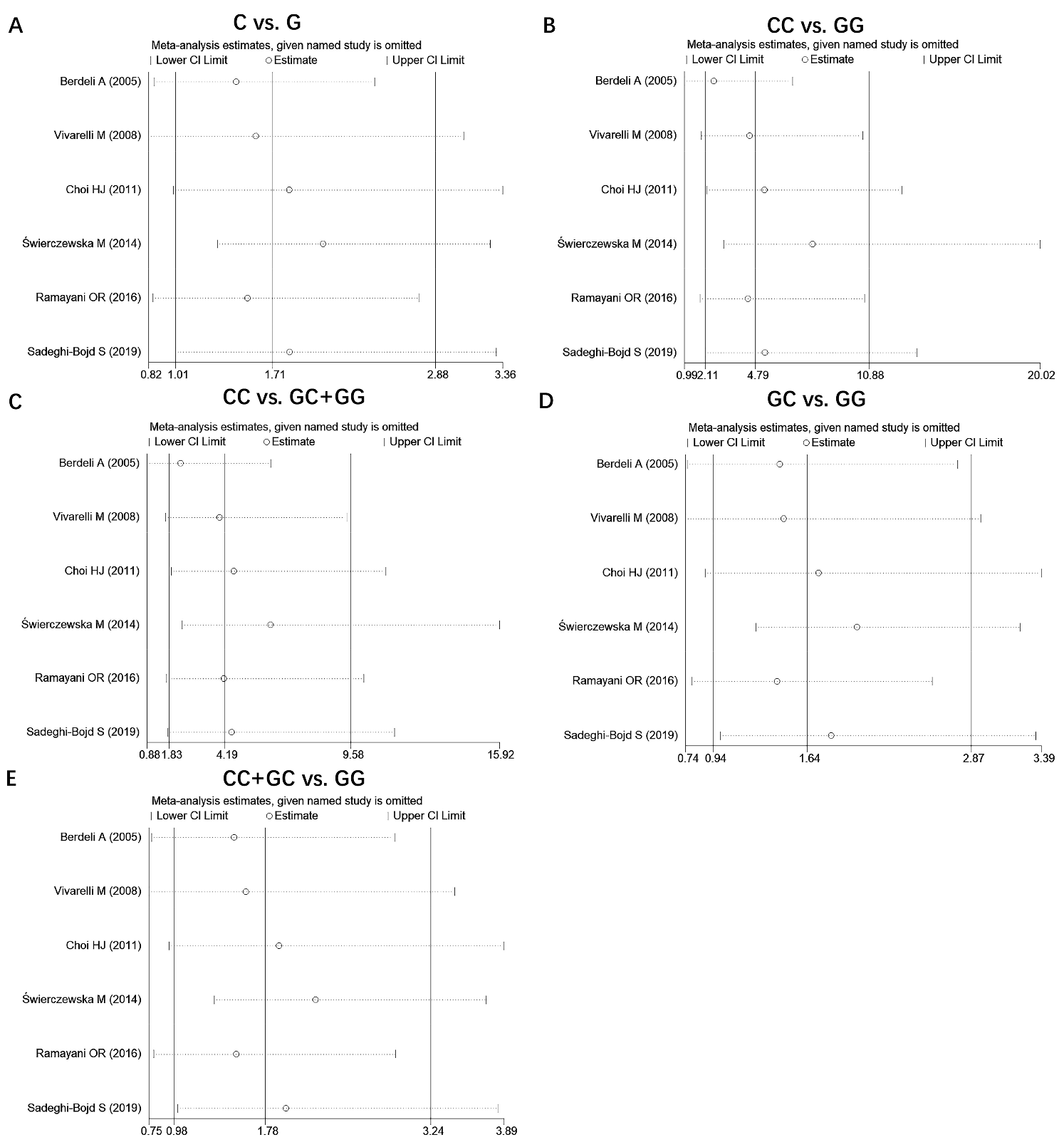

Supplement: Supplementary file 1 [file Data_Sheet_1.docx]
